# (1-Adamant­yl)(3-amino­phen­yl)methanone

**DOI:** 10.1107/S1600536811046009

**Published:** 2011-11-05

**Authors:** Michal Rouchal, Marek Nečas, Robert Vícha

**Affiliations:** a Department of Chemistry, Faculty of Technology, Tomas Bata University in Zlin, Nám. T. G. Masaryka 275, Zlín,762 72, Czech Republic; b Department of Chemistry, Faculty of Science, Masaryk University, Kamenice 5, Brno-Bohunice, 625 00, Czech Republic

## Abstract

In the crystal sructure of the title compound, C_17_H_21_NO, the mol­ecular packing is stabilized by inter­molecular N—H⋯O hydrogen bonds and additional weak N—H⋯π inter­actions, forming chains that propagate along the *b* axis. Conjugation of the carbonyl group and the benzene ring is rather attenuated due to a twisting of the carbonyl group from the plane of the benzene ring [torsion angle = 27.1 (2)°].

## Related literature

For recent reviews of the biological activity of some adamantane-bearing compounds, see: Ahrén (2009 ▶); Ginsberg (2010 ▶); Lagoja & De Clercq (2008 ▶). For the structures of similar adamantylated aromatic amines, see: Rouchal *et al.* (2009 ▶, 2011 ▶).
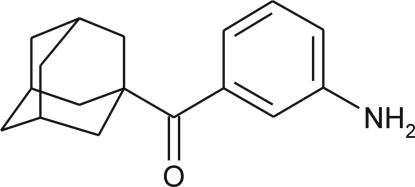


## Experimental

### 

#### Crystal data

C_17_H_21_NO
*M* *_r_* = 255.35
Orthorhombic, 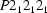

*a* = 6.4644 (1) Å
*b* = 8.1978 (3) Å
*c* = 25.1760 (5) Å
*V* = 1334.17 (6) Å^3^
*Z* = 4
Mo *K*α radiation
μ = 0.08 mm^−1^
*T* = 120 K
0.30 × 0.30 × 0.20 mm

#### Data collection

Oxford Diffraction Xcalibur Sapphire2 diffractometer
Absorption correction: multi-scan (*CrysAlis RED*; Oxford Diffraction, 2009 ▶) *T*_min_ = 0.998, *T*_max_ = 1.000
15931 measured reflections
1672 independent reflections
1531 reflections with *I* > 2σ(*I*)
*R*_int_ = 0.016

#### Refinement

*R*[*F* ^2^ > 2σ(*F* ^2^)] = 0.030
*wR*(*F* ^2^) = 0.073
*S* = 1.04
1672 reflections
180 parameters
H atoms treated by a mixture of independent and constrained refinement
Δρ_max_ = 0.21 e Å^−3^
Δρ_min_ = −0.17 e Å^−3^

### 

Data collection: *CrysAlis CCD* (Oxford Diffraction, 2009 ▶); cell refinement: *CrysAlis RED* (Oxford Diffraction, 2009 ▶); data reduction: *CrysAlis RED*; program(s) used to solve structure: *SHELXS97* (Sheldrick, 2008 ▶); program(s) used to refine structure: *SHELXL97* (Sheldrick, 2008 ▶); molecular graphics: *ORTEP-3* (Farrugia, 1997 ▶) and *Mercury* (Macrae *et al.*, 2008 ▶); software used to prepare material for publication: *SHELXL97*.

## Supplementary Material

Crystal structure: contains datablock(s) global, I. DOI: 10.1107/S1600536811046009/pk2356sup1.cif

Structure factors: contains datablock(s) I. DOI: 10.1107/S1600536811046009/pk2356Isup2.hkl

Supplementary material file. DOI: 10.1107/S1600536811046009/pk2356Isup3.cml

Additional supplementary materials:  crystallographic information; 3D view; checkCIF report

## Figures and Tables

**Table 1:** Hydrogen-bond geometry (Å, °) *Cg*1 is the centroid of the C12–C17 ring.

*D*—H⋯*A*	*D*—H	H⋯*A*	*D*⋯*A*	*D*—H⋯*A*
N1—H1*B*⋯O1^i^	0.91 (3)	2.10 (3)	3.003 (2)	168 (2)
N1—H1*A*⋯*Cg*1^ii^	0.90 (3)	2.54 (3)	3.316 (18)	144 (2)

Symmetry codes: (i) 
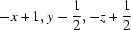
; (ii) 
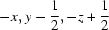
.

**Fig. 1.**
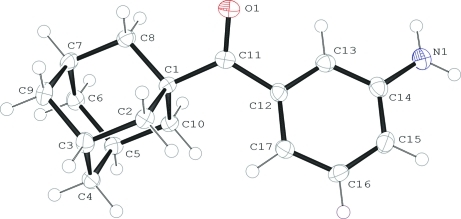
A thermal ellipsoid plot (50% probability) of the asymmetric unit. H-Atoms are shown as small spheres at arbitrary radii.

**Fig. 2.**
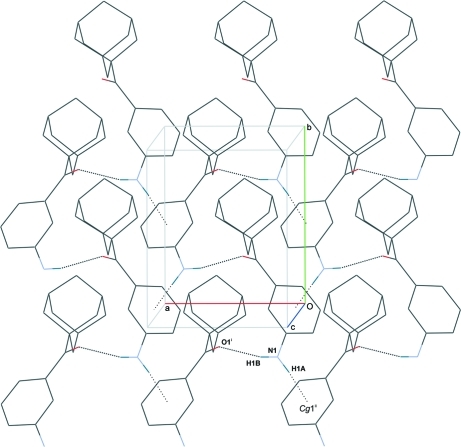
The H-bonded chains of the molecules of title compound arranged parallel to the b-axis. H-atoms have been omitted (except for those participating in H-bonds) for clarity. Cg1 is centre of gravity of C12—C17. Symmetry codes: (i) -x + 1, y - 1/2, -z + 1/2; (ii) -x, y - 1/2, -z + 1/2.

## supplementary crystallographic information

### Comment

The adamantane cage represents a widely used substituent in medicinal chemistry. A large number of adamantylated biologically active compounds have been described in the literature. For example, they act as tuberculostatics (Ginsberg, 2010), anti-influenza virus agents (Lagoja & De Clercq, 2008) and as type 2 diabetes medications (Ahrén, 2009). The title molecule belongs to the family of newly prepared adamantane-bearing aromatic amines as promising building blocks for drugs modification.

The asymmetric unit of the title compound consists of a single molecule (Fig. 1). The benzene ring is essentially planar with a maximum deviation from the best plane being 0.025 (16) Å for C14. The adamantane cage consists of three fused cyclohexane rings in classical chair conformations, with C—C—C angles varying within the range 107.40 (12)–110.82 (13)°. The torsion angles describing an arrangement of adamantane scaffold, benzene ring and carbonyl bridge C1–C11–C12–C13 and C2–C1–C11–C12 are 151.40 (15) and 78.16 (17)°, respectively. The molecules are linked into chains parallel to the *b*-axis by N1–H1A···O1 hydrogen bonds (Fig. 2, Table 1).  The crystal packing is further stabilized by intermolecular N–H···π interactions.

### Experimental

(1-Adamantyl)(3-nitrophenyl)methanone (450 mg, 1.65 mmol) was dissolved in 47 cm^3^ of warm methanol and 7 cm^3^ of hydrochloric acid/water (1/1, *v*/*v*) was carefully added. Into the refluxed and well stirred mixture, portions of an iron powder (207 mg, 3.71 mmol) were added successively. The reaction was stopped when TLC indicated the consumption of all starting material. The mixture was diluted with 5% solution of sodium hydroxide (40 cm^3^) and extracted several times with diethyl ether. Combined organic layers were washed with brine, dried over sodium sulfate and evaporated in vacuum. The desired product was obtained after the purification of crude material using column chromatography (silica gel; petroleum ether/ethyl acetate, 1/1, *v*/*v*) as a colourless crystalline powder (371 mg, 88%, mp 370–373 K). The crystal used for data collection was grown by spontaneous evaporation from deuterochloroform at room temperature.

### Refinement

All carbon bound H atoms were placed at calculated positions with distances of 1.00 Å (R_3_CH), 0.99 Å (R_2_CH_2_) and 0.95 Å (C_sp2_H), and were refined as riding with their *U*_iso_ set to 1.2*U*_eq_ of the respective carrier atoms. Nitrogen bound H atoms were located in a difference Fourier map and refined isotropically. In the absence of anomalous scattering, Friedel pairs were merged.

### Crystal data

**Table:**

C_17_H_21_NO	*D*_x_ = 1.271 Mg m^−^^3^
*M**_r_* = 255.35	Melting point: 372 K
Orthorhombic, *P*2_1_2_1_2_1_	Mo *K*α radiation, λ = 0.71073 Å
Hall symbol: P 2ac 2ab	Cell parameters from 10578 reflections
*a* = 6.4644 (1) Å	θ = 3.2–27.2°
*b* = 8.1978 (3) Å	µ = 0.08 mm^−^^1^
*c* = 25.1760 (5) Å	*T* = 120 K
*V* = 1334.17 (6) Å^3^	Block, colourless
*Z* = 4	0.30 × 0.30 × 0.20 mm
*F*(000) = 552	

### Data collection

**Table:**

Oxford Diffraction Xcalibur Sapphire2 diffractometer	1672 independent reflections
Radiation source: fine-focus sealed tube	1531 reflections with *I* > 2σ(*I*)
graphite	*R*_int_ = 0.016
Detector resolution: 8.4353 pixels mm^-1^	θ_max_ = 27.3°, θ_min_ = 3.2°
ω scan	*h* = −8→8
Absorption correction: multi-scan (*CrysAlis RED*; Oxford Diffraction, 2009)	*k* = −10→5
*T*_min_ = 0.998, *T*_max_ = 1.000	*l* = −32→32
15931 measured reflections	

### Refinement

**Table:**

Refinement on *F*^2^	Primary atom site location: structure-invariant direct methods
Least-squares matrix: full	Secondary atom site location: difference Fourier map
*R*[*F*^2^ > 2σ(*F*^2^)] = 0.030	Hydrogen site location: inferred from neighbouring sites
*wR*(*F*^2^) = 0.073	H atoms treated by a mixture of independent and constrained refinement
*S* = 1.04	*w* = 1/[σ^2^(*F*_o_^2^) + (0.0342*P*)^2^ + 0.3516*P*] where *P* = (*F*_o_^2^ + 2*F*_c_^2^)/3
1672 reflections	(Δ/σ)_max_ < 0.001
180 parameters	Δρ_max_ = 0.21 e Å^−^^3^
0 restraints	Δρ_min_ = −0.17 e Å^−^^3^

### Special details

**Table:**

Geometry. All e.s.d.'s (except the e.s.d. in the dihedral angle between two l.s. planes) are estimated using the full covariance matrix. The cell e.s.d.'s are taken into account individually in the estimation of e.s.d.'s in distances, angles and torsion angles; correlations between e.s.d.'s in cell parameters are only used when they are defined by crystal symmetry. An approximate (isotropic) treatment of cell e.s.d.'s is used for estimating e.s.d.'s involving l.s. planes.
Refinement. Refinement of *F*^2^ against ALL reflections. The weighted *R*-factor *wR* and goodness of fit *S* are based on *F*^2^, conventional *R*-factors *R* are based on *F*, with *F* set to zero for negative *F*^2^. The threshold expression of *F*^2^ > 2σ(*F*^2^) is used only for calculating *R*-factors(gt) *etc*. and is not relevant to the choice of reflections for refinement. *R*-factors based on *F*^2^ are statistically about twice as large as those based on *F*, and *R*- factors based on ALL data will be even larger.

### Fractional atomic coordinates and isotropic or equivalent isotropic displacement parameters (Å^2^)

**Table:**

	*x*	*y*	*z*	*U*_iso_*/*U*_eq_	
O1	0.4200 (2)	0.29596 (16)	0.21048 (4)	0.0309 (3)	
N1	0.1688 (3)	−0.2699 (2)	0.23832 (7)	0.0344 (4)	
C1	0.3647 (2)	0.3819 (2)	0.12126 (6)	0.0170 (3)	
C2	0.1811 (2)	0.5018 (2)	0.11371 (6)	0.0190 (3)	
H2A	0.0525	0.4396	0.1069	0.023*	
H2B	0.1613	0.5667	0.1465	0.023*	
C3	0.2246 (2)	0.6165 (2)	0.06674 (6)	0.0214 (4)	
H3	0.1048	0.6922	0.0619	0.026*	
C4	0.2554 (3)	0.5155 (2)	0.01592 (6)	0.0220 (4)	
H4A	0.1285	0.4522	0.0083	0.026*	
H4B	0.2818	0.5892	−0.0145	0.026*	
C5	0.4390 (2)	0.3989 (2)	0.02303 (6)	0.0202 (3)	
H5	0.4585	0.3339	−0.0102	0.024*	
C6	0.6351 (2)	0.4984 (2)	0.03404 (6)	0.0226 (4)	
H6A	0.6638	0.5718	0.0037	0.027*	
H6B	0.7547	0.4242	0.0385	0.027*	
C7	0.6041 (3)	0.5995 (2)	0.08489 (6)	0.0213 (4)	
H7	0.7323	0.6638	0.0923	0.026*	
C8	0.5599 (2)	0.4852 (2)	0.13178 (6)	0.0204 (3)	
H8A	0.5401	0.5504	0.1645	0.025*	
H8B	0.6799	0.4121	0.1374	0.025*	
C9	0.4214 (3)	0.7162 (2)	0.07774 (7)	0.0234 (4)	
H9A	0.4488	0.7911	0.0477	0.028*	
H9B	0.4028	0.7825	0.1103	0.028*	
C10	0.3976 (3)	0.2828 (2)	0.06965 (6)	0.0184 (3)	
H10A	0.5164	0.2078	0.0741	0.022*	
H10B	0.2730	0.2164	0.0621	0.022*	
C11	0.3283 (2)	0.2699 (2)	0.16894 (6)	0.0193 (3)	
C12	0.1869 (2)	0.1237 (2)	0.16726 (6)	0.0190 (3)	
C13	0.2376 (3)	−0.0037 (2)	0.20158 (6)	0.0215 (3)	
H13	0.3620	0.0023	0.2216	0.026*	
C14	0.1096 (3)	−0.1401 (2)	0.20729 (6)	0.0232 (4)	
C15	−0.0785 (3)	−0.1424 (2)	0.17955 (6)	0.0252 (4)	
H15	−0.1727	−0.2299	0.1847	0.030*	
C16	−0.1272 (3)	−0.0174 (2)	0.14471 (6)	0.0246 (4)	
H16	−0.2532	−0.0220	0.1254	0.030*	
C17	0.0044 (3)	0.1143 (2)	0.13749 (6)	0.0217 (3)	
H17	−0.0290	0.1973	0.1126	0.026*	
H1A	0.068 (4)	−0.338 (3)	0.2494 (10)	0.058 (8)*	
H1B	0.286 (4)	−0.257 (3)	0.2582 (9)	0.048 (7)*	

### Atomic displacement parameters (Å^2^)

**Table:**

	*U*^11^	*U*^22^	*U*^33^	*U*^12^	*U*^13^	*U*^23^
O1	0.0383 (7)	0.0326 (7)	0.0219 (6)	−0.0106 (7)	−0.0096 (6)	0.0026 (5)
N1	0.0409 (10)	0.0300 (9)	0.0324 (8)	−0.0094 (9)	−0.0087 (8)	0.0115 (8)
C1	0.0158 (7)	0.0174 (7)	0.0177 (7)	−0.0016 (7)	−0.0002 (6)	−0.0010 (7)
C2	0.0167 (7)	0.0182 (8)	0.0221 (7)	0.0007 (8)	0.0027 (6)	−0.0010 (7)
C3	0.0161 (8)	0.0194 (8)	0.0287 (8)	0.0030 (7)	0.0011 (6)	0.0047 (7)
C4	0.0187 (7)	0.0246 (9)	0.0226 (8)	−0.0025 (8)	−0.0022 (6)	0.0061 (7)
C5	0.0191 (8)	0.0233 (8)	0.0181 (7)	0.0000 (8)	0.0009 (6)	−0.0015 (7)
C6	0.0164 (7)	0.0259 (9)	0.0255 (8)	−0.0009 (8)	0.0035 (6)	0.0041 (7)
C7	0.0164 (7)	0.0220 (9)	0.0254 (8)	−0.0046 (8)	0.0003 (6)	0.0002 (7)
C8	0.0176 (7)	0.0222 (8)	0.0215 (7)	−0.0026 (8)	−0.0016 (6)	0.0000 (7)
C9	0.0235 (9)	0.0186 (8)	0.0283 (8)	−0.0039 (8)	0.0042 (7)	0.0017 (7)
C10	0.0183 (8)	0.0179 (8)	0.0191 (7)	0.0001 (7)	−0.0008 (7)	−0.0019 (6)
C11	0.0192 (7)	0.0204 (8)	0.0183 (7)	0.0023 (7)	−0.0005 (6)	−0.0011 (7)
C12	0.0211 (7)	0.0199 (8)	0.0160 (7)	−0.0014 (7)	0.0026 (6)	0.0000 (7)
C13	0.0214 (7)	0.0244 (8)	0.0186 (7)	0.0002 (8)	−0.0007 (6)	−0.0013 (7)
C14	0.0300 (9)	0.0221 (9)	0.0174 (7)	−0.0010 (8)	0.0022 (7)	0.0005 (7)
C15	0.0288 (9)	0.0225 (9)	0.0245 (8)	−0.0081 (8)	0.0027 (7)	−0.0014 (7)
C16	0.0238 (8)	0.0270 (9)	0.0230 (8)	−0.0053 (8)	−0.0035 (7)	−0.0026 (7)
C17	0.0250 (8)	0.0209 (8)	0.0193 (7)	−0.0004 (8)	−0.0026 (6)	0.0009 (7)

### Geometric parameters (Å, °)

**Table:**

O1—C11	1.2208 (19)	C6—H6B	0.9900
N1—C14	1.375 (2)	C7—C9	1.530 (2)
N1—H1A	0.90 (3)	C7—C8	1.534 (2)
N1—H1B	0.91 (2)	C7—H7	1.0000
C1—C11	1.529 (2)	C8—H8A	0.9900
C1—C8	1.543 (2)	C8—H8B	0.9900
C1—C10	1.547 (2)	C9—H9A	0.9900
C1—C2	1.553 (2)	C9—H9B	0.9900
C2—C3	1.537 (2)	C10—H10A	0.9900
C2—H2A	0.9900	C10—H10B	0.9900
C2—H2B	0.9900	C11—C12	1.508 (2)
C3—C4	1.537 (2)	C12—C13	1.395 (2)
C3—C9	1.538 (2)	C12—C17	1.400 (2)
C3—H3	1.0000	C13—C14	1.398 (2)
C4—C5	1.535 (2)	C13—H13	0.9500
C4—H4A	0.9900	C14—C15	1.402 (2)
C4—H4B	0.9900	C15—C16	1.385 (2)
C5—C6	1.532 (2)	C15—H15	0.9500
C5—C10	1.535 (2)	C16—C17	1.386 (2)
C5—H5	1.0000	C16—H16	0.9500
C6—C7	1.538 (2)	C17—H17	0.9500
C6—H6A	0.9900		
			
C14—N1—H1A	117.0 (16)	C9—C7—H7	109.4
C14—N1—H1B	117.0 (15)	C8—C7—H7	109.4
H1A—N1—H1B	120 (2)	C6—C7—H7	109.4
C11—C1—C8	108.68 (12)	C7—C8—C1	110.82 (13)
C11—C1—C10	111.41 (13)	C7—C8—H8A	109.5
C8—C1—C10	108.66 (12)	C1—C8—H8A	109.5
C11—C1—C2	111.02 (12)	C7—C8—H8B	109.5
C8—C1—C2	107.40 (12)	C1—C8—H8B	109.5
C10—C1—C2	109.56 (12)	H8A—C8—H8B	108.1
C3—C2—C1	109.96 (12)	C7—C9—C3	109.08 (13)
C3—C2—H2A	109.7	C7—C9—H9A	109.9
C1—C2—H2A	109.7	C3—C9—H9A	109.9
C3—C2—H2B	109.7	C7—C9—H9B	109.9
C1—C2—H2B	109.7	C3—C9—H9B	109.9
H2A—C2—H2B	108.2	H9A—C9—H9B	108.3
C2—C3—C4	109.58 (14)	C5—C10—C1	109.89 (13)
C2—C3—C9	109.77 (13)	C5—C10—H10A	109.7
C4—C3—C9	109.22 (13)	C1—C10—H10A	109.7
C2—C3—H3	109.4	C5—C10—H10B	109.7
C4—C3—H3	109.4	C1—C10—H10B	109.7
C9—C3—H3	109.4	H10A—C10—H10B	108.2
C5—C4—C3	109.79 (13)	O1—C11—C12	117.21 (14)
C5—C4—H4A	109.7	O1—C11—C1	119.54 (15)
C3—C4—H4A	109.7	C12—C11—C1	123.23 (13)
C5—C4—H4B	109.7	C13—C12—C17	119.21 (15)
C3—C4—H4B	109.7	C13—C12—C11	115.82 (14)
H4A—C4—H4B	108.2	C17—C12—C11	124.75 (15)
C6—C5—C4	109.22 (13)	C12—C13—C14	121.58 (15)
C6—C5—C10	109.66 (13)	C12—C13—H13	119.2
C4—C5—C10	109.91 (13)	C14—C13—H13	119.2
C6—C5—H5	109.3	N1—C14—C13	120.85 (16)
C4—C5—H5	109.3	N1—C14—C15	120.94 (17)
C10—C5—H5	109.3	C13—C14—C15	118.19 (15)
C5—C6—C7	109.25 (13)	C16—C15—C14	120.17 (16)
C5—C6—H6A	109.8	C16—C15—H15	119.9
C7—C6—H6A	109.8	C14—C15—H15	119.9
C5—C6—H6B	109.8	C15—C16—C17	121.31 (16)
C7—C6—H6B	109.8	C15—C16—H16	119.3
H6A—C6—H6B	108.3	C17—C16—H16	119.3
C9—C7—C8	109.21 (13)	C16—C17—C12	119.34 (15)
C9—C7—C6	109.85 (13)	C16—C17—H17	120.3
C8—C7—C6	109.60 (14)	C12—C17—H17	120.3

### Hydrogen-bond geometry (Å, °)

**Table:**

*Cg*1 is the centroid of the C12–C17 ring.

**Table:**

*D*—H···*A*	*D*—H	H···*A*	*D*···*A*	*D*—H···*A*
N1—H1B···O1^i^	0.91 (3)	2.10 (3)	3.003 (2)	168 (2)
N1—H1A···Cg1^ii^	0.90 (3)	2.54 (3)	3.316 (18)	144 (2)

Symmetry codes: (i) −*x*+1, *y*−1/2, −*z*+1/2; (ii) −*x*, *y*−1/2, −*z*+1/2.
